# Congenital Ocular Findings in an Antillean Manatee (*Trichechus manatus manatus*)

**DOI:** 10.1002/vms3.70989

**Published:** 2026-05-21

**Authors:** Fernanda Loffler Niemeyer Attademo, Radan Elvis Matias de Oliveira, Fábia de Oliveira Luna, Helena Gurjão Pinheiro do Val, Lucas Inácio dos Santos Melo, Lisandra Hannah Shttoves da Silva, Bruna Bezerra, Fabrício Bezerra de Sá

**Affiliations:** ^1^ Programa de Pós‐Graduação em Ciência Animal/PPGCA Universidade Federal Rural do Semiárido/UFERSA Mossoró Rio Grande do Norte Brazil; ^2^ Centro Nacional de Pesquisa e Conservação de Mamíferos Aquáticos/CMA Instituto Chico Mendes de Conservação da Biodiversidade/ICMBio Ilha de Itamaracá Pernambuco Brazil; ^3^ Departamento de Zoologia, Programa de Pós‐Graduação em Biologia Animal/PPBA, Laboratório de Ecologia Comportamento e Conservação/LECC Universidade Federal de Pernambuco/UFPE Recife Pernambuco Brazil; ^4^ Universidade Federal Rural de Pernambuco Recife Pernambuco Brazil

**Keywords:** cataract, dysplasia, eye, manatee, retina

## Abstract

The West Indian manatee (*Trichechus manatus manatus*), a sirenian species critically endangered throughout its range, possesses unique ocular adaptations to aquatic life, including reduced eyes, vascularized corneas and an anteriorly displaced lens. We aimed to document the first known case of congenital bilateral cataract associated with retinal dysplasia in a stranded manatee calf. We evaluated a manatee neonate calf, about 4 days old, kept in the Center for Rehabilitation of the National Center for Research and Conservation of Aquatic Mammals on Itamaracá Island, Pernambuco State, Brazil. Clinical signs included impaired postural behaviour, failure to avoid obstacles and inability to locate food independently. Ophthalmologic evaluation revealed absent menace response and glare reflex, supporting the hypothesis of visual impairment. Ultrasonographic imaging confirmed bilateral lens opacity and retinal folding consistent with retinal dysplasia, without signs of inflammation or persistent hyperplastic primary vitreous. Although congenital cataracts may regress in terrestrial mammals, follow‐up examination 2 years later indicated persistence and slight progression of lens opacity in this case. These findings expand current understanding of congenital ocular disorders in aquatic mammals and underscore the importance of early, thorough clinical assessments for rescued individuals. Furthermore, the presence of such conditions may complicate rehabilitation and release efforts, emphasizing the need for additional research into the aetiology, progression and conservation impact of congenital diseases in small, vulnerable populations of threatened species.

**Summary:**

- First documented case of congenital cataract and retinal dysplasia in an Antillean manatee.
- Visual impairment strongly correlated with reduced exploratory behaviour and is possible cause of stranding.

## Graphical abstract

This study reports the first documented case of bilateral congenital cataract associated with retinal dysplasia in an Antillean manatee (*Trichechus manatus manatus*) calf. Ophthalmologic examination and ocular ultrasonography confirmed lens opacity at the posterior pole, which correlated with behavioural deficits consistent with visual impairment. A 2‐year follow‐up revealed persistence and slight progression of the cataract, whereas behavioural responses improved, highlighting the value of ultrasonography as a non‐invasive tool for diagnosis and monitoring during rehabilitation.

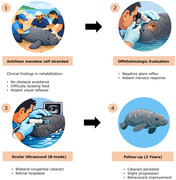

## 1 Introduction

The Antillean manatee (*Trichechus manatus manatus*) is an herbivorous aquatic mammal that inhabits coastal and estuarine areas in the Northern and Northeastern Brazilian regions and is among the most threatened marine mammals nationally (Luna et al. 2010; Domit et al. 2023). Aquatic mammals have specific adaptations for underwater vision, including light‐sensing mechanisms like emmetropia; pupil shapes that regulate light entry, mainly rod photoreceptors with fewer cones; a spherical lens; corneal design allowing acute vision in both air and water; a blue reflective optic tapetum; specific ciliary muscles in the eye and thickened corneas (Waller 1992; Mass and Supin 2007; Fasick and Robinson 2016). They also have unique neural characteristics like large ganglion cells separated by wide intercellular spaces, enabling efficient processing of visual information (Mass and Supin 2007; Fasick and Robinson 2016). Hence, those species exhibit a diverse range of structural modifications that enable them to inhabit aquatic environments with distinct physical properties, a topic widely discussed in studies aimed at understanding their ecology and evolution (Reidenberg 2007).

The external ocular anatomy of *T. manatus manatus* comprises diminutive eyeballs with diameters from 13 to 19 mm, the presence of a protective nictitating internal membrane and is devoid of eyelids. Internally, the eyeballs differ from those of other aquatic mammals, being relatively spherical on both sides and deep, and with shallow anterior chambers (Hartman 1971; Cohen et al. 1982; Mass and Supin 2007; Oliveira et al. 2026). The small crystalline has an oval format and is anteriorly displaced and is attached to the eyeballs by the ciliary corpus. Unlike most mammals, manatees’ corneas are vascularized by a sparse blood supply network (Harper et al. 2005; Mass and Supin 2007).

Although Antillean manatees are aquatic, the species’ visual quality differs above and below the water. Underwater, visual acuity tends towards ametropia, a condition characterized by incorrect focusing of light on the retina, whilst in contact with air (i.e., above water), vision is predominantly myopic (Mass and Supin 2007). Nonetheless, their eyes are dynamic out of water, which involves pupil‐muscle closure, significantly increasing the complexity of the procedure and making it challenging to perform. This short communication has, then, the objective of describing the findings of visual complications in a female Antillean manatee calf in Brazil.

## 2 Case Presentation

The manatee neonate calf, about 4 days old, named Wind (registered under no. 01S0112/353), was found on 27 April 2022, stranded on the beach of Macapá in the city of Luís Corrêa, Piauí State (−2.91158 S, −41.44758 W). On 30 April, it was transported by aeroplane to the Center for Rehabilitation of the National Center for Research and Conservation of Aquatic Mammals on Itamaracá Island, Pernambuco State, Brazil. There, the calf was kept in a rounded fibre pool of 5.54 m^3^ (2.9 *Ø* × 0.9 m), which had three steps at different heights (bottom and intermediate: always underwater; high: exposed above water). The pool was supplied twice a day, in the mornings and evenings, with marine water previously treated with sodium hypochlorite.

In the first days of life, the calf presented clinical signs of constipation due to sand ingestion at the stranding. However, once the treatment for digestive issues was completed, the calf remained mostly immobile, with minimal movement and little exploratory behaviour. The animal only moved when its body was in contact with the pool's borders, and its face was preferably near the pool steps. It was also observed that the calf did not avoid either object introduced into the pool or the steps, consistently hitting its rostrum. During bottle feeding or when solid food was available on the surface of the water, the animal did not show a tendency to approach the objects, which had to be manually guided into the calf's mouth by caretakers. Those perceptions led to the suspicion of a blind animal. Apart from visual issues, we observed increased hearing sensitivity, leading the animal to be more stressed by noises near the pool. However, this hypothesis was not further tested.

To confirm the blindness diagnosis, objects were carefully introduced in the pool, avoiding noises and animal disturbance. In none of the attempts did the animal deviate from the object, remaining static in front of it until its removal. This behaviour persisted for 6 months, and after this period, there appeared to be a gradual reduction in the inability to recognize objects, without any sign of ocular modification.

**FIGURE 1:**
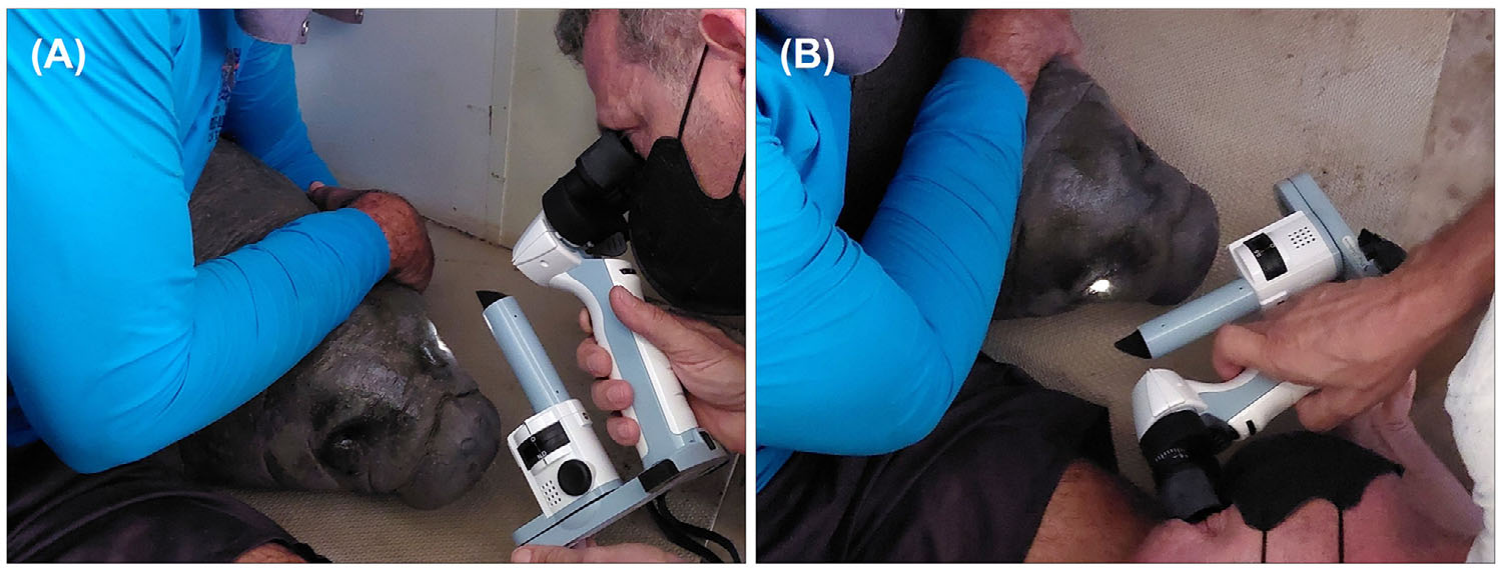
Ophthalmic examination of a calf Antillean manatee (*Trichechus manatus manatus*): (A) examination of the left eye and (B) examination of the right eye.

First, the Visual Glare Test (Figure 1) was performed, which revealed a negative glare, slow or absent, meaning that once exposed to light, the animal did not have an immediate reaction of closing the eyes. Moreover, Wind did not react to threatening tests, indicating the lack of awareness of movement in the surroundings, supporting the hypothesis of a visual impedance and a cataract condition. Although the retina showed a regular pattern of irrigation via a blood vessel network extending throughout its entire extent, we observed displacement from the sclera due to retinal overgrowth. This condition causes the retina to fold in some areas, characterizing a clinical form of retinal dysplasia, known to have a congenital origin. There was no observed persistence of hyperplastic primary vitreous (PHPV), only a light irregularity of the crystalline due to the cataract, which consists of an evolution of the nuclear cataract. Although it is a congenital condition, there are no signs of the cataract being hereditary.

**FIGURE 2:**
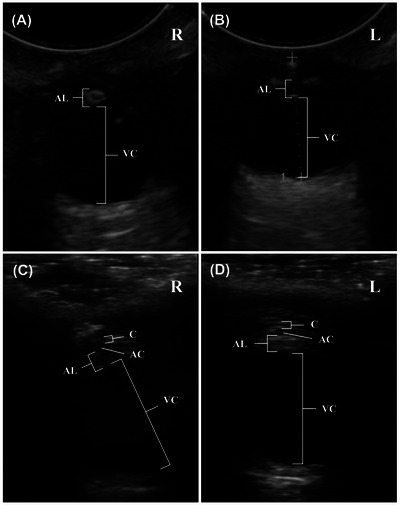
Ultrasonographic findings of congenital cataract in the Antillean manatee (*Trichechus manatus manatus*) using B‐mode. (A and B) Ultrasonographic examination of the right and left eyes, performed at 2 months of age with a microconvex transducer, shows increased echogenicity of the lenses. (C and D) Ultrasound examination of the right and left eyes, performed at 2 years and 3 months of age using a linear transducer, reveals that the lenses remain echogenic, indicating persistence of the cataract over time. AC, anterior chamber; AL, axial lens; C, corneal; L, left eye; R, right eye; VC, vitreous chamber.

After the initial diagnosis of the cataract, an ultrasound image exam (US) was performed, aiming to investigate the presence of any other potential morphological anomalies in Wind's eye. For this procedure, we used a topical anaesthetic made of proxymetacaine 0.5% in both eyes. During the exam, we observed opacities in the posterior lens of both eyes, without any signs of inflammation, consistent with a clinical diagnosis of bilateral congenital cataracts (Figure 2A,B). Two years and 1 month as the first exam, a new ultrasound exam was performed. In this exam, we noted the persistence of bilateral cataracts and an increase in lens echogenicity compared with the previous exam, suggesting a discrete progression of the opacity at the lens (Figure 2C,D).

## 3 Discussion and Conclusions

Ophthalmologic pathologies have been described in different species of aquatic mammals, such as cornea lesions in dolphins (*Tursiops truncatus*) (Colitz et al. 2016); ceratite in California sea‐lions (*Zalophus californianus*), Steller sea‐lions (*Eumetopias jubatus*) and seals (Colitz et al. 2010); and cataract in Northern sea‐elephants (*Mirounga angustirostris*) (Filer et al. 2003). However, to our knowledge, there is no record of ophthalmic pathologies in Sirenian species, making this the first report of both cataract and retinal dysplasia in the Antillean manatee.

The results of the ophthalmic exams suggest a direct relationship between reduced visual acuity and the calf's postural behaviour, as the difficulty in recognizing the pool environment can explain reduced mobility and exploratory behaviours. Additionally, we hypothesize that the visual deficit may have been one of the causes of its stranding, as once separated from its mother, the calf would be unable to self‐orient in the sea. This hypothesis is supported mainly by the calf's early age at rescue.

A cataract is a clinical condition characterized by opacity in the crystalline lens that creates a physical barrier to the passage of light, preventing the formation of an image on the retina (Salomão et al. 2009). In the present communication, a manatee calf was diagnosed with a congenital cataract. Some studies in other species suggest this alteration is a consequence of intrauterine infections, such as toxoplasmosis or congenital hypothyroidism due to thyroid dysfunction, among other causes (Melamed et al. 2010; Razavi et al. 2012; Farassat et al. 2021). A study conducted with children by Arun et al. (2007) observed that congenital cataract resulting from toxoplasmosis was associated with other intraocular pathologies, such as retinal dysplasia. Nonetheless, the congenital cataract had been observed to regress spontaneously, with total recovery of visual acuity, without surgical intervention, in domestic dogs under 1‐year‐old (Gelatt 1975). *Toxoplasma gondii* infection may be subclinical or manifest with diverse clinical signs. Attademo et al. (2016) detected *T. gondii* in Brazilian manatees, with all reported cases exhibiting subclinical presentation. In contrast, congenital transmission has been documented in domestic species, such as dogs and cats, and is associated with retinochoroiditis and other ocular abnormalities. However, the serologic exam for *T. gondii* in this calf failed to detect antibodies to the parasite, thereby rejecting the hypothesis of this infection.

Dog breeds, such as the German Shepherd, exhibit predisposition to this congenital ocular condition (Zubrický and Trbolová 2022). However, as this represents the first documented case in manatees to the authors’ knowledge, it is premature to conclude that it constitutes a population‐specific trait at the site where the calf was rescued. Genetic factors represent the leading cause of congenital cataracts. It is not possible to determine the primary cause of this finding. However, because the calf was found in this condition at only a few days of age, a congenital origin of unknown aetiology is suggested. Genetic and environmental studies of manatee populations at the calves’ site of origin are recommended to assess the circulation of infectious agents, genetic factors or consanguinity that may have contributed to these findings.

The retina, a structure localized at the posterior aspect of the eyeball, is responsible for image formation, which is subsequently transmitted to the central nervous system (Hartman 1971). Contrary to cataract, retinal dysplasia is irreversible (Jakobiec et al. 2011) but would not affect Wind's visual quality if vision is recovered. Nevertheless, it is important to highlight that, given the absence of stimulus in the occipital lobe, the area of the brain in which the image is processed, it is possible that even with the regression of the cataract, the calf may not be able to react to visual stimulus. In this case, the blindness would be characterized as cortical blindness, the most common cause of congenital visual deficiency in children (Piña‐Garza and James 2019; Kirshner 2021). Another issue is that, due to the manatees’ mass, their prolonged time out of water during the 30 days required for post‐surgery procedures is impractical, making it impossible to remove the cataract surgically.

The originality of this study for the Sirenian order prevents comparability with other cases, particularly regarding the age at which cataract regression could occur. Since the 18‐month‐old, Wind has not presented visual difficulties and developed typically for its age, reaffirming the initial prognosis that cataracts in manatees could be reversible, as in other species. A discussion on the possible causes of these alterations and the implications for wild manatees is necessary. Moreover, population studies on Antillean manatees and the species’ vulnerability to diseases and congenital conditions must also be considered in conservation plans.

The longitudinal ophthalmic evaluation of this Antillean manatee revealed a condition compatible with bilateral congenital cataract, with slow progression and no associated signs of ocular inflammation. Until the present case study, threats to Antillean manatees comprised environmental factors, pollution and disease, but not congenital cataracts and associated ocular conditions within their populations.

The initial diagnosis was supported by ultrasonographic findings demonstrating lens opacity restricted to the posterior pole of both lenses, a morphological feature often associated with congenital forms of the condition in a wide range of aquatic mammals. Congenital cataracts in aquatic mammals have been less frequently reported than in terrestrial species. They are usually associated with specific morphological features, such as lens opacity at the posterior pole of the crystalline lens. In other wild mammals, genetic mutations have been associated with the formation of congenital cataracts that affect crystalline protein function, leading to a similar opacity to that observed in this case (You et al. 2021; Bai et al. 2021).

The slow but progressive increase in crystalline opacity observed over 25 months, without any evidence of other ocular anomalies, such as retinal dysplasia or vitreous degeneration, is characteristic of nonsyndromic primary congenital cataracts. This pattern is supported by similar findings in humans and other animals, in which congenital cataracts that tend to progress insidiously are not followed by other ocular structural imperfections (Graw 2004; Shiels and Hejtmancik 2017). Genetic mutations that affect crystalline proteins—such as crystalline or connexins—are usually associated with nonsyndromic congenital cataracts and can lead to gradual increases in crystalline opacity over time (Li et al. 2020). The absence of additional ocular pathology supports the primary nature of the lens fault rather than a secondary or syndromic process (Shiels and Hejtmancik 2017). Regular follow‐ups in cases like this are essential, as even a smooth progression can eventually affect visual capacity, and the course of the progression can vary accordingly to the genetic cause. In general, Wind's clinical observations and imaging exams are consistent with the natural history of nonsyndromic primary congenital cataracts described in other mammalian species (Graw 2004; Shiels and Hejtmancik 2017).

Ultrasonographic follow‐up demonstrated a discrete yet progressive increase in lens echogenicity, whereas the opacity remained localized to the posterior region of the crystalline lens. Notably, this morphological progression did not parallel a worsening of functional outcomes. On the contrary, the calf exhibited a gradual improvement in behavioural responses consistent with visual perception, including increased environmental exploration and object recognition. The predominantly turbid aquatic environment inhabited by Antillean manatees, combined with the species’ reliance on non‐visual sensory modalities, may reduce functional dependence on high visual acuity and mitigate the clinical impact of partial or posterior cataracts. Manatees possess highly innervated vibrissae, including perioral tactile hairs, along with refined somatosensory perception and effective underwater hearing, which play a central role in spatial orientation, environmental exploration and feeding behaviour (Sarko et al. 2007; Gaspard et al. 2013; Moore et al. 2021; Cook et al. 2025). These sensory adaptations may compensate for early visual deficits and help explain the gradual improvement in functional behaviour observed in this calf, despite the persistence of lens opacity detected by ultrasonography.

In this case study, we highlight that maintaining ocular morphological structure over time reinforces the stability of the conditions and provides essential support for the clinical management and conservation of the Antillean manatee, especially for individuals under extensive human care. This attendance represents an unprecedented contribution to ophthalmology in aquatic mammals, highlighting the utility of ultrasonographic examinations as a non‐invasive tool for ocular monitoring in manatees, particularly in the context of rehabilitation and pre‐release assessment.

The present study reports, for the first time, a case of cataract in Antillean manatees, raising awareness of Sirenians’ susceptibility to developing visual anomalies and of how this condition can impact wild populations. However, systematic monitoring of the case plays a crucial role in determining the condition's evolution and the decision regarding its possible release to the wild, or not, even with cataract regression. Additionally, more studies are needed to evaluate the prevalence and functional impact of ocular pathologies in manatees, as well as the possible causes of a congenital issue in the species, with direct implications for developing strategies to conserve *T. manatus manatus* in the wild and throughout the rehabilitation process.

Ultrasonographic identification of the opacity at the posterior pole was demonstrated to be an effective tool for diagnosing congenital cataracts in manatees. This finding highlights the importance of genetic and morphological evaluations in identifying possible congenital diseases and their implications for species conservation.

## Author Contributions

**Fernanda Loffler Niemeyer Attademo**: conceptualization, methodology, investigation, formal analysis, writing – original draft, writing – review and editing. **Radan Elvis Matias de Oliveira**: methodology, investigation, formal analysis, writing – original draft, writing – review and editing. **Fábia de Oliveira Luna**: conceptualization, data curation, validation, funding acquisition, project administration, writing – original draft. **Helena Gurjão Pinheiro do Val**: investigation, formal analysis, writing – original draft. **Lucas Inácio dos Santos Melo**: investigation, formal analysis. **Lisandra Hannah Shttoves da Silva**: investigation, formal analysis, funding acquisition. **Bruna Bezerra**: methodology, formal analysis, writing – original draft. **Fabrício Bezerra de Sá**: conceptualization, methodology, investigation, formal analysis, supervision, funding acquisition, writing – original draft.

## Funding

The authors have nothing to report. The Article Processing Charge (APC) for the publication of this research was funded by the Coordenação de Aperfeiçoamento de Pessoal de Nível Superior ‐ Brasil (CAPES) (code 001)

## Ethics Statement

The authors have nothing to report.

## Conflicts of Interest

The authors declare no conflicts of interest.

## Data Availability

The data that support the findings of this study are available from the corresponding author upon reasonable request.
